# SmTAL-9, a Member of the *Schistosoma mansoni* Tegument Allergen-Like Family, Is Important for Parasite Survival and a Putative Target for Drug/Vaccine Development

**DOI:** 10.3389/fimmu.2022.889645

**Published:** 2022-07-12

**Authors:** Wilma Patrícia de Oliveira Santos Bernardes, Isabela Thamara Xavier Dutra, Rosiane Aparecida da Silva-Pereira, Marina Moraes Mourão, Cristina Toscano Fonseca

**Affiliations:** ^1^ Grupo de Pesquisas em Biologia e Imunologia de Doenças Infecciosas e Parasitárias, Instituto René Rachou, Fundação Oswaldo Cruz, Belo Horizonte, Brazil; ^2^ Grupo de Pesquisas em Helmintologia e Malacologia Médica, Instituto René Rachou, Fundação Oswaldo Cruz, Belo Horizonte, Brazil

**Keywords:** Smtal-9, immunization, *Schistosoma mansoni*, RNA interference, protection

## Abstract

The tegument of *Schistosoma mansoni* is involved in essential functions for parasite survival and is known to stimulate immune responses in pre-clinical vaccine trials. Smtal-9, a member of the tegument-allergen-like (TAL) family, is one of the components of the tegument of schistosomula recognized by sera from immunized and protected mice. In this work, we assessed the role of Smtal-9 in parasite survival using the RNAi approach. Also, we cloned and expressed a recombinant form of Smtal-9 and evaluated its ability to induce protection in mice. *Smtal-9* knockdown did not impact parasite survival *in vitro*, but significantly decreased schistosomula size. Additionally, significant reduction in both parasite and egg burdens were observed in mice inoculated with *Smtal-9*-knockdown schistosomula. Immunization using the Smtal-9 as an antigen conferred partial protection against challenge infection. Overall, our results indicate that Smtal-9 is a candidate target for drug and/or vaccine development due to its important role in parasite biology and survival.

## Introduction

The schistosome tegument represents one of the interfaces between parasite and host, and is involved in essential functions of parasite biology, such as nutrition, sensory perception, evasion of the immune system and osmoregulation (1). Additionally, it has been demonstrated that the tegument itself, as well as several tegument proteins, such as SmTSP-2, Sm29, Stomatin-like protein-2 and Sm200 are able to induce protective immunity when used in vaccine formulations in pre-clinical trials (2–7).

Among the various parasite tegument proteins, Smtal-1, a member of the tegument-allergen-like (TAL) family, has previously been associated with resistance to infection/reinfection. Smtal-1 has been pointed as the major target of an IgE response that is associated with resistance to reinfection in individuals living in endemic areas for schistosomiasis (8). Additionally, when Smtal-1 was used as antigen in a vaccine formulation, partial resistance against infection was observed in mice (9). Besides the recognition of Smtal-1, IgE antibodies from the sera of individuals living in endemic areas for schistosomiasis also recognize other members from the TAL family, such as Smtal-2 and Smtal-5 (10), but it is not known if this recognition is able to induce some level of resistance to parasite infection.

The *Schistosoma* TAL family includes 13 proteins that are associated with the worm tegument, Smtal-1 to 13 (10–12). These proteins are characterized by the presence of one or two EF-hand motifs in the N-terminal region of the protein, and a dynein light chain (DLC)-1 domain in the C-terminal region (10, 13, 14). These characteristics, together with the demonstration that Smtal–3 can bind the *Schistosoma mansoni* dynein (SmDCL), suggest that proteins from TAL family may be involved in vesicle transport in the parasite tegument (12, 14).

Smtal–9 (Smp_077310.1) is a member of the TAL family which is predicted to be expressed throughout the parasite life cycle, though most abundantly in eggs and adult worms (10). This member of the TAL family was recently biochemically characterized, and the study described its ability to form homodimers and to bind Mn^2+^, Mg^2+^, Ca^2+^, as well as to interact with the calmodulin antagonists CPZ, W7 and TFP, suggesting its involvement in Ca^2+^–dependent signal transduction (15). In another proteomics study, analyzing the schistosomula tegument preparation (Smteg), a protein spot recognized by sera from mice immunized with Smteg (5) was identified as a putative *S. mansoni* tegumental protein (XP_018655674.1) (unpublished data). According to the online databank WormBase ParaSite, this protein is a product of gene *Smtal–9* (Smp_077310). In this study, we aimed to evaluate the role of Smtal–9 in parasite survival, and to investigate its ability to induce a protective immune response in mice, because of the key role of antibodies against tegument proteins in the protective immunity induced by vaccination (16). Our data indicates that RNAi–mediated *Smtal–9* knockdown in schistosomula negatively impacts on parasite survival within the host, resulting in a reduction in the worm burden, as well as in the number of eggs trapped in the liver and intestine of infected mice. In addition, a transient reduction in *Smtal–9* expression resulted in small–sized parasites *in vitro*. The immune response triggered by a prime–boost immunization protocol using Smtal–9 as antigen, also generated significant levels of protection (19.3 to 28.6%), measured as a reduction in parasite burden. Overall, our data indicate that Smtal–9 is a candidate target for drug and vaccine development.

## Methods

### Animals and Parasite Culture

In this study, all procedures involving animals were approved by the Ethics Committee of Animal Use of the Fundação Oswaldo Cruz (FIOCRUZ) under licenses LW26/12 and LW22/16. Female Balb/c and C57BL–6 mice aged 6–8 weeks were used and obtained from the Institute René Rachou (IRR)/FIOCRUZ animal facility. *Schistosoma mansoni* cercariae from the LE strain were obtained from the mollusc facility “Lobato Paraense” of IRR/FIOCRUZ by exposing infected *Biomphalaria glabrata* snails to artificial light for about 2 hours to induce shedding. LE *Schistosoma mansoni*/Belo Horizonte was obtained by Dr. J. Pellegrino in Belo Horizonte, Minas Gerais, Brazil, in 1965 from an infected human patient (17). The line is maintained at the mollusck rearing facility “Lobato Paraense” of IRR/FIOCRUZ by passage through *B. glabrata* and mice or hamsters

RNAi trials were performed using cercariae mechanically transformed into schistosomula according to Milligan and Jolly (17) with modifications. Briefly, cercariae solution was distributed in 50ml conical tubes that were placed on ice for one hour to sediment the cercariae. Then, after centrifugation at 218 xg for 3 minutes at 4°C, the supernatant was removed. The final 7ml solution, enriched with cercariae, was passed through a 22 gauge needle until all tails were removed. After transformation, schistosomula were sedimented in a conic tube, resuspended in Minimal Essential Medium (MEM) and incubated a 37°C for one hour. After that, parasites were placed in Glasgow Minimum Essential Medium (GMEM) (Merck) supplemented with 0.1% lactalbumin (Merck), 5% Schneider’s Insect Medium (Merck), 20 mM of HEPES (Merck), 0.1% glucose (Vetec), 0.5% MEM vitamin solution (Gibco, Thermo Fisher Scientific, USA), 2% inactivated fetal bovine serum (Gibco, Thermo Fisher Scientific, USA), 0.2 mM of triiodothyronine (Merck), 1 mM of hydrocortisone (Merck), 0.5 mM of hypoxanthine (Merck), and 1% penicillin/streptomycin (Gibco, Thermo Fisher Scientific, USA) and incubated in a biological oxygen demand (BOD) incubator at 37°C under 5% CO_2_ (18).

### *Smtal–9* ds–RNA Synthesis and Gene Expression Assessment

The sequence of the primers used for double–stranded RNA (dsRNA) synthesis and quantitative real–time PCR (RT–qPCR) were designed using the Primer 3 program (v.0.4.0) (bioinfo.ut.ee/primer3–0.4.0) and purchased from Integrated DNA Technologies Inc. (Coralville, IA). The primers used in the synthesis of the dsRNA carried a T7 promoter sequence added to their 5′–end. The *Smtal–9* mRNA (XM.018790318) containing 354 bp was amplified by PCR using the following primers: forward 5–taatacgactcactatagggAAAACAAGCGCGGGAATTA–3′ and reverse 5–taatacgactcactatagggGCAGAACATGGTGTTTTCCA–3. Total RNA from schistosomula was used as the template for cDNA synthesis and *Smtal–9* amplification. A 360 bp fragment of the *green–fluorescent protein* (*GFP*) gene was used as a nonspecific control for the RNAi and was amplified using the pCRII–GFP plasmid vector (Thermo Fisher Scientific, USA) as a template and the following primers: *gfp*_dsRNA_Forward, 5–taatacgactcactatagggGTGTTCAATGCTTTGCGAGA–3, and *gfp*_dsRNA_Reverse, 5–taatacgactcactatagggCTTTTCGTTGGGATCTTTCG–3’.

The dsRNAs were synthetized using PCR products and T7 RiboMAX™ Express RNAi System Kit (Promega, USA), according to the manufacturer instructions. A Nanodrop Spectrophotometer ND–1000 (Thermo Fisher Scientific, USA) was used to determine the dsRNA concentration. The integrity of the dsRNA was determined by analysis on a 1% agarose gel.

Approximately 12,000 schistosomula were exposed to 200 nM of dsRNA (s*mtal–9 or gfp*) in a 6–well plate containing supplemented GMEM medium (3,000 parasites/ml). As an additional control, parasites were incubated in supplemented GMEM only. Cultures were incubated for up to ten days after dsRNA exposure at 37°C, 5% CO_2_ and 95% humidity. Approximately 3,000 schistosomula between days four to seven post–dsRNA–exposure were removed from the culture for *Smtal–9* relative gene expression analysis using RT–qPCR. TRIzol Reagent (Thermo Fisher Scientific, USA) was used for the RNA extractions, as recommended by the manufacturer. The extracted RNA was quantified using the Qubit 2.0 Fluorometer (Thermo Fischer Scientific, USA) after removing residual DNA from the extracted RNA with Turbo DNase (2 U/*μ*l) (Thermo Fisher Scientific, USA). The ImProm–II™ Reverse Transcription System (Promega, USA) was used to produce the cDNA for the RT–qPCR. After standardization to determine the appropriate primer concentration, the RT–qPCR experiments were performed using 200 nM of primers that amplify a 126 bp *Smtal–9* fragment (forward 5′–CTCGTTTTTGGACGCTTTTT– 3′ and reverse 5′–CGACGAAGAAATGTTTCTGGA–3′). The *smgapdh* (Smp–056970) gene was used as an endogenous normalization control. A fragment of 53 bp of *smgapdh* was amplified using 900 mM of the following primers (forward 5′–TCGTTGAGTCTACTGGAGTCTTTACG–3′ and reverse 5′–AATATGAGCCTGAGCTTTATCAATGG–3′). The transcript levels of *Smtal–9* were evaluated using the relative 2^–^ *^ΔΔ^* ^Ct^ method (19) and calculated as a percentage of difference compared to the nonspecific *gfp*–treated controls and untreated parasites. PCR reactions were performed using a ViiA 7 (Thermo Fisher Scientific, USA) at the Real–Time PCR Facility/RPT09D PDTIS/René Rachou Institute/FIOCRUZ MG.

### *In Vitro* and *In Vivo* RNAi Assay Using Schistosomula

For the RNAi assay, schistosomula were separately treated with either *Smtal–9–* or *gfp–*dsRNA or supplemented medium lacking dsRNA. Schistosomula were examined for phenotypic changes using an inverted fluorescence microscope (Axio Observer, Carl Zeiss), as previously described (19). Parasite viability was evaluated by incubating approximately 100 schistosomula with 5 *μ*g/ml propidium iodide (Sigma–Aldrich), and the stained parasites were observed and counted using a fluorescence microscope with a 544 nm filter (Carl Zeiss). Data from three independent assays using 100 schistosomula er assay were analyzed.

To analyze the role of Smtal–9 in parasite’s definitive host, after schistosomula exposure to *Smtal–9–* or *gfp–dsRNA* for 4 days, approximately 270 larvae were subcutaneously inoculated into Balb–c mice. An additional control group was inoculated with untreated schistosomula. Before infection of mice with dsRNA–treated and untreated parasites the *Smtal–9* transcript levels were determined by RT–qPCR to confirm knockdown. Three independent experiments were performed using 10–12 mice in each group per experiment. Fifty days after subcutaneous inoculation, adult worms were recovered by perfusion of the hepatic portal system from mice (20). The liver and gut from mice were weighed and digested with 10% KOH solution for subsequent determination of the egg number per gram of organ. The experimental approach used to investigate the role of Smtal–9 in parasite development and survival is illustrated in the **Supplementary Figure 1**.

### *In Silico* Analysis and Smtal–9 Modelling

The *S. mansoni* Smtal–9 (access number: XP_018655674.1) protein had its molecular weight and isoelectric point predicted using the compute pi/MW tool (https://web.expasy.org/compute_pi/). The protein amino acid sequence was analyzed using SignalP5.0 (https://services.healthtech.dtu.dk/service.php?SignalP–5.0) to predict signal peptides in its sequence. Also, SOSUI Engine version 1.11 (http://harrier.nagahama–i–bio.ac.jp/sosui/sosui_submit.html) and TMHMM (http://www.cbs.dtu.dk/services/TMHMM/) were used to predict transmembrane domains and protein solubility. WolfPSORT (https://wolfpsort.hgc.jp/ ) was used to predict cellular localization. Post transcriptional changes were predicted using NetNGlyc (http://www.cbs.dtu.dk/services/NetNGlyc/), NetOGlyc (http://www.cbs.dtu.dk/services/NetOGlyc/) and Big–PI prediction (https://mendel.imp.ac.at/gpi/gpi_server.html). The XP_018655674.1 amino acid sequence was compared with the sequences from the WormBase ParaSite databank using the BlastP (BLAST – Basic Local Alignment Search Tool) algorithm (https://parasite.wormbase.org/tools/blast), with the default parameters of this algorithm. Multiple sequence alignments of XP_018655674.1, Smp–077310.1, Smp–077310.2 and Smp–324770.1 were performed using Clustal Omega (http://www.clustal.org/omega/).

T cell epitope prediction was performed using NetMHCII 2.3 (https://services.healthtech.dtu.dk/service.php?NetMHCII–2.3) using default parameters and selecting H2–IaB and H2–IaD to predict epitopes that strongly bind to C57BL–6 and BALB–c MHCII molecules, respectively. B cell linear epitopes were predicted using the BepiPred (https://services.healthtech.dtu.dk/service.php?BepiPred–2.0) with default parameters.

The amino acid sequence of the *S. mansoni* Smtal–9 recombinant protein (**Figure 3**) was submitted to build a three–dimensional homology model by automated comparative modeling using Protein Homology/analogy Recognition Engine V 2.0 (Phyre^2^) using Intensive Modelling Mode (21). The resulting structure for the recombinant protein was predicted using seven different templates (d3e2ba1, d1cmia, c6zywM, c7kznP, c5x2eA, c5fx0A and c5x,2dA) with >90% confidence with 90% sequence coverage (173 residues).

### Recombinant Antigen Preparation

A synthetic gene containing the coding region sequence for Smtal–9 (access number (XP_018655674.1) was designed using ApE software (www.apesoftware.com ), optimized for expression in bacteria and mammals. The synthetic gene was purchased from IDT Technologies, and contained restriction sites for *BamH*I (GGATCC) at the 5′ end and *Xho*I (CTCGAG) followed by *Age*I (ACCGGT) at the 3′ end. These restriction sites were used to subclone the synthetic gene into the pET28a TEV bacterial expression vector (22) or into the mammalian expression vector pcDNA3.1 V5/His A (Thermo Fisher Scientific, USA). The constructs were inserted into *Escherichia coli* BL21 and DH5α strains. Antibiotic–resistant bacteria clones were selected, and the presence of the construct and the correct orientation of the *Smtal–9* open reading frame were confirmed by Sanger sequencing. The pcDNA 3.1 V5/His A and pcDNA 3.1 V5/His A/*Smtal–9* plasmidial DNA were purified using EndoFree Plasmid Giga Kit (Qiagen), according to manufacturer’s instructions. Recombinant Smtal–9 expression in fusion with the 6–histidine–tag was induced by the addition of 1 mM isopropyl–B–D–galactopyranoside to the LB medium. Bacteria were maintained at 37°C for 4 hours at 150 rpm, and then, after a centrifugation step (4800 x g for 10 min) the pellet was resuspended in PBS 0.15 M pH 7.2 containing 1 mM phenylmethylsufonyl fluoride (PMSF) (Sigma Aldrich) and 0.2 mM EDTA (Sigma Aldrich) and lysed by 5 cycles of sonication (5 x 30 seconds using 30% output followed by 1 min on ice). After the last sonication step, the cell lysate was centrifuged at 8000 x g for 10 min, and the pellet resuspended in a buffer containing 20 mM NaH_2_PO_4_, 0.5 M NaCl, 30 mM imidazole and 0.9% N–Lauroylsarcosine sodium salt (Sigma Aldrich) and used for protein purified using a His Trap HP column (GE Life Science) in an AKTA FPLC equipment (GE Life Science). Elution was performed using a linear gradient from 0 to 100% of 20 mM Na_2_HPO_4,_ 0.5 M NaCl, pH 7.4 with 500 mM of imidazole. Recombinant protein was dialyzed against a 0.15 M PBS solution for 24 hours and quantified using the BCA Protein Assay Kit (Thermo Scientific Pierce, Rockford, IL, USA) before its use in experimental protocols. Protein quality and purity were analyzed using 15% SDS–PAGE, and its recognition by an anti–His (C–term) HRP antibody (Novex, life technology) was assessed by a western blot assay. Briefly, proteins were separated using a 15% SDS–PAGE and transferred to a nitrocellulose membrane at 100 V for one hour. The membrane was blocked over night with 1X TBS containing 0.05% of Tween 20 (TBST) and 5% of powdered skimmed milk. After three washing steps with TBST for 10 minutes each, the membrane was incubated with an anti–His HRP conjugated antibody diluted 1:5000 in TBST plus 3% of powdered skimmed milk for one hour. The membrane was submitted to three additional washing steps, and the recognition of the His–tag in the recombinant protein was detected using ECL™ Prime Western Blotting Detection Reagent (GE Healthcare). The western blot result was acquired using the Amersham Imager 600 equipment (GE).

### Prime and Boost Immunization Protocol

A prime–boost immunization protocol was performed using female C57BL–6 mice (12 animals per group) using Smtal–9 as antigen. Five days before the beginning of the immunization protocol, mice were inoculated with 50 μl of cardiotoxin (10 μM) in each quadriceps muscle. At day 0, mice received 100 μg (50 μg/quadriceps muscle) of pcDNA 3.1V5/HIS or pcDNA 3.1V5/HIS/*Smtal9.* Fifteen days after the prime immunization with the DNA vaccine, mice received two doses of the protein vaccine containing 25 μg of rSmtal–9/dose inoculated subcutaneously in a 15–day interval between them. As a control, two groups (pcDNA 3.1V5/HIS + Saline CFA/IFA and pcDNA 3.1V5/HIS/*Smtal9* + Saline CFA/IFA) were inoculated with a saline solution instead of the recombinant protein. Both saline and rSmtal–9 were formulated with complete Freund`s adjuvant at the first boost and incomplete Freund`s adjuvant at the second boost. The immunization regimen and formulations for each experimental group are summarized in **Table 1** and illustrated in **Supplementary Figure 2**. Fifteen days after the last boost, mice were percutaneously infected with approximately 100 *S. mansoni* cercariae (LE strain) and fifty days after challenge infection mice were perfused as previously described (20) for the recovery of worms from the mesenteric veins. The liver and intestine from mice were also obtained, and the number of eggs trapped in these organs was determined as previously described (23). Two independent immunization trials were performed.

**Table 1:** Immunization regimen and formulations.

Experimental group	Prime formulation	1^st^ boost formulation	2^nd^ boost formulation
pcDNA 3.1V5/HIS + Saline CFA/IFA	100 μg pcDNA 3.1V5/HIS	Saline + 100 μl of complete Freund`s adjuvant (CFA)	Saline + 100 μl of incomplete Freund`s adjuvant (IFA)
pcDNA 3.1V5/HIS + rSmtal–9 CFA/IFA	100 μg pcDNA 3.1V5/HIS	25 μg of rSmtal–9 + 100μl of complete Freund`s adjuvant (CFA)	25 μg of rSmtal–9 + 100μl of complete Freund`s adjuvant (CFA)
pcDNA 3.1V5/HIS/*Smtal9* + Saline CFA/IFA	100 μg pcDNA 3.1V5/HIS/*Smtal9*	Saline + 100μl of complete Freund`s adjuvant (CFA)	Saline + 100 μl of incomplete Freund`s adjuvant (IFA)
pcDNA 3.1V5/HIS/*Smtal9* + rSmtal9 CFA/IFA	100 μg pcDNA 3.1V5/HIS/*Smtal9*	25 μg of rSmtal–9 + 100μl of complete Freund`s adjuvant (CFA)	25 μg of rSmtal–9 + 100μl of complete Freund`s adjuvant (CFA)

### Statistical Analysis

The data were analyzed using GraphPad Prism 8.0 (Graph–Pad Software, San Diego, CA, United States). The distribution of the data was analyzed using the Shapiro–Wilk normality test, followed by the Brown–Forsythe or Bartlett`s test to assess the variance homogeneity. When the results from the different groups showed significant differences in variance, the data were analyzed pair by pair using the Student’s *t* test or the Mann–Whitney *U* test using the alpha value adjusted according to the number of comparisons made between the groups. When the variances did not differ significantly between groups, one–way ANOVA or the Kruskal–Wallis test, followed by Tukey or Dunn’s multiple comparison tests, was used in statistical analysis using a 95% a confidence level. Two–way ANOVA was used to calculate statistically significant differences between groups and over time in the viability assay.

## Results

### *Smtal–9* Expression Is Essential for Parasite Development and Survival *In Vivo*

**Figure 1:**
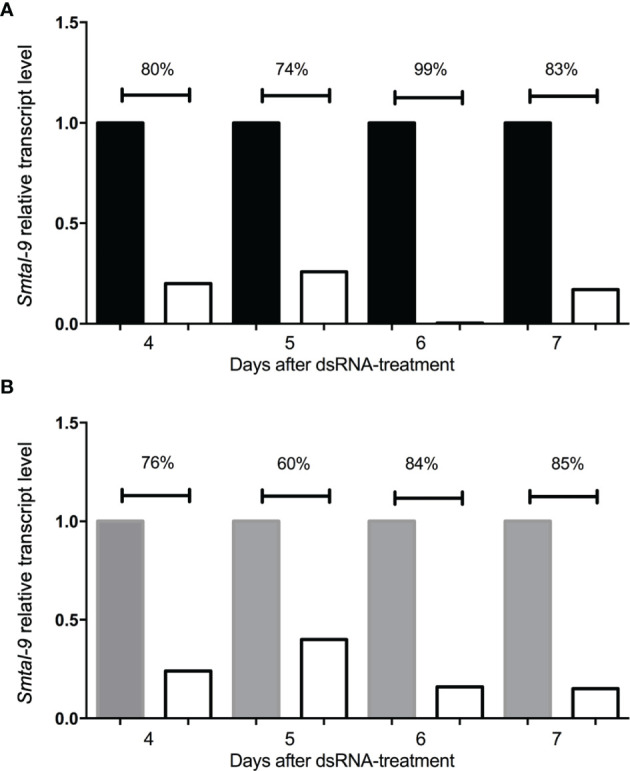
Kinetics of *smtal–9* transcript levels after schistosomula dsRNA exposure for 7 days. Graphical depiction of the relative *smtal–9* transcript levels and their comparison between *smtal–9* dsRNA (white bars in both charts) exposed schistosomula and two different control groups: untreated parasites (black bars in **A**) or parasites treated with *gfp*–specific dsRNA (grey bars in **B**). Transcripts were evaluated between the fourth to the seventh day after dsRNA exposure. Transcript levels of *smtal–9* were evaluated by RT–qPCR. Percentage of reduction of the *smtal–9* transcript levels in s*mtal–9*–dsRNA treated parasites was calculated in comparison to the transcript level observed in untreated **(A)** or *gfp*–dsRNA **(B)** treated parasites.

**Figure 2:**
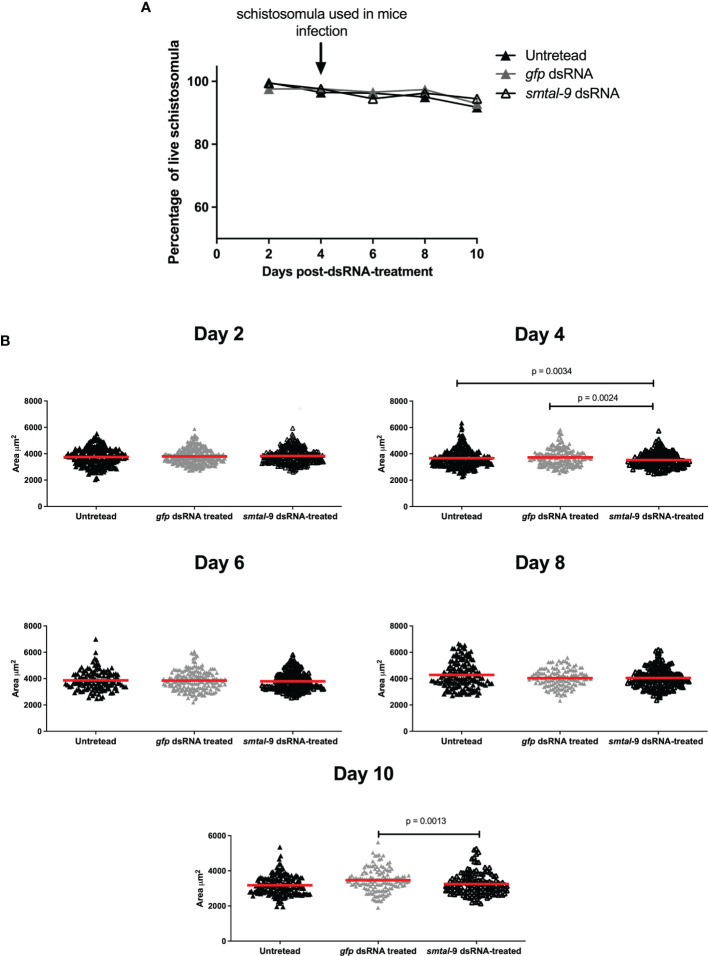
*In vitro* impact of *smtal–9* knockdown on parasite survival and size. Schistosomula were treated with either *smtal–9*–dsRNA (open triangle), *gfp*–dsRNA (grey triangle) or untreated (closed triangle). **(A)** Parasite viability was evaluated every two days after exposure to dsRNA until day 10 post–exposure. The percentage of live parasites was calculated from three independent replicate experiments and differences between groups were determined using one–way ANOVA. **(B)** Parasite size was calculated for parasites recovered from the cultures of each group every two days until the 10^th^ day post–dsRNA–exposure. The size of each parasite is represented in the graphs, with the red line representing the mean parasite area in each group. Significant differences between groups are indicated on the graphs and were determined using the Mann–Whitney *U* test using an adjusted α value.

A RNAi assay was performed to evaluate the role of Smtal–9 in parasite biology. Double–stranded RNA was used to target *Smtal–9* transcripts in schistosomula. The dsRNA used to knockdown *Smtal–9* targets the two protein isoforms of Smtal–9 (Smp–077310.1 and Smp–077310.2) as well as Smp–324770 in RNAi assays (**Supplementary Figure 3A**). The transcript levels were determined in schistosomula at days 4, 5, 6 and 7 post–exposure to dsRNA in comparison to two negative controls: schistosomula exposed to *gfp*–dsRNA and untreated schistosomula. Decreased levels of transcripts were observed in schistosomula exposed to *Smtal–9*–dsRNA at days 4 (80%–76%), 5 (74%–60%), 6 (99%–84%) and 7 (83%–85%) post–exposure when compared to untreated (**Figure 1A**) and *gfp*–dsRNA treated parasites, respectively (**Figure 1B**), indicating that the RNAi assay succeeded in knocking down *Smtal–9* gene expression. The impact of *Smtal–9* knockdown on schistosomula survival and morphology was evaluated *in vitro* at days 2, 4, 6, 8 and 10 after parasite exposure to specific and nonspecific dsRNA. Parasite treatment with either dsRNA did not impact parasite survival *in vitro*, since no significant differences in parasite viability was observed between dsRNA–exposed and unexposed parasites (**Figure 2A**). Parasite area was also evaluated after exposure to *Smtal–9* specific and nonspecific dsRNA. Four days after treatment with dsRNA, a significant decrease in parasite size was observed in schistosomula treated with *Smtal–9*–dsRNA in comparison to both negative controls (nonspecific *gfp*–dsRNA and untreated parasites) (**Figure 2B**). This difference in parasite size was not observed at days 6 and 8 post–treatment and was only observed between *Smtal–9–*dsRNA–treated and *gfp*–dsRNA–treated parasites on the 10^th^ day (**Figure 2B**). To evaluate the impact of *Smtal–9* expression in parasite survival in the vertebrate host, mice were inoculated with *Smtal–9–*dsRNA–treated schistosomula four days after exposure to dsRNA, when differences in the size–phenotype were observed in *in vitro* cultured parasites. Regardless of the control group, in all assays, there was a significant reduction in parasite burden (56.5 – 71.4%) in mice inoculated with *Smtal–9* knocked–down schistosomula (**Table 2**). Reduced number of eggs trapped in the liver (53.8 – 70%) and in the intestine (64% – 81%) were also observed in mice inoculated with *Smtal–9* knocked–down parasites (**Table 2**).

**Table 2:** Worm burden recovery from mice infected with schistosomula knocked–down *Smtal–9* expression.

	Transcript reduction (%)	Worm burden recoveryMean ± SD	% reduction^*^&^^	Egg/gram of liverMean ± SD	% reduction^#&^	Egg/gram of intestineMean ± SD	% reduction^#&^
**Trial 1**							
Untreated		23 ± 16		20119 ± 9920		8956 ± 7722	
*Smtal9–dsRNA–*treated	48.0%	10 ± 7	56.5% (p = 0.0226)	8437 ± 5774	58.0% (p=0.0026)	2853 ± 2161	68.0% (p=0.0161)
**Trial 2**							
Untreated		18 ± 11		19797 ± 8823		5815 ± 4790	
*Smtal9–dsRNA–*treated	86.4%	7 ± 5	61.1% (p = 0.0226)	5959 ± 6017	70.0% (p = 0.0016)	1118 ± 1488	81.0% (p = 0.0041)
**Trial 3**							
*GFP–dsRNA*–treated		7 ± 5		6247 ± 4191		1541 ± 1458	
*Smtal9–dsRNA–*treated	76.0%	2 ± 2	71.4% (p = 0.0178)	2883 ± 1870	53.8% (p = 0.0281)	552 ± 473	64.0% (ns)

*Reduction of in the total number of worms compared to control group.

^#^Reduction of in the total number of eggs in tissue (liver and intestine) compared to control group.

^&^Statistically significant differences were determined using the Student’s t test or the Mann–Whitney U test.

### A Vaccine Formulation Containing Smtal–9 Reduces Parasite Burden in Mice

**Figure 3:**
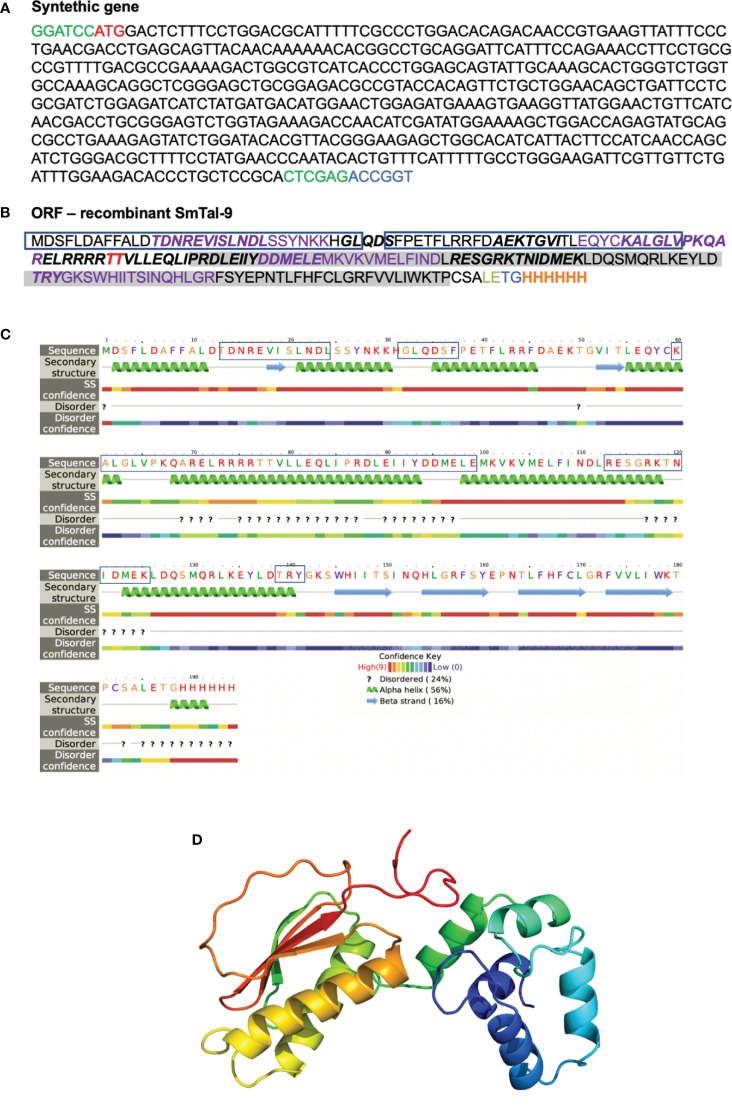
*In silico* analysis of rSmTAL–9. **(A)** Nucleotide sequence of the synthetic gene encoding rSmTAL–9 with the added *BamH*I, and *Xho*I restriction sites in green at the 3’ and 5’ ends, respectively, and the *Age*I restriction site in blue at the 5’ end. The start codon is indicated in red. **(B)** The open reading frame sequence of rSmTAL–9. The linear B cell epitopes predicted by Bepipred is indicated in bold. The amino acids highlighted in purple indicate epitopes that bind the H2–IaD haplotype. The amino acid in red represents a predicted site for O–glycosylation. Boxes highlight the EF–hand motif, while the DLC motif is highlighted in grey, and the His–tag is indicated in orange. **(C)** Secondary structure within rSmTAL–9 amino acid sequence obtained using Phyre in intensive mode. Boxes highlight predicted linear B cell epitopes by Bepipred. **(D)** Three–dimensional model of the rSmTAL–9 obtained using Phyre2 in intensive mode.

Since the RNAi functional assay suggested an important role for Smtal–9 in parasite survival within the definitive host, we assessed whether an immune response triggered against this tegument protein would induce protection in mice. *In silico* analysis of the Smtal–9 protein sequence (XP_018655674.1) used to produce the recombinant protein used in this study (**Figures 3A, B**) indicated that Smtal–9 is a soluble cytoplasmic protein with predicted 22kDa and 6.44 isoelectric point. No signal peptide, transmembrane domain nor GPI–anchor site was predicted in this sequence. A BLASTp search of the above sequence against trematode proteins from the parasite WormBase ParaSite databank demonstrated that the sequence used to generate the recombinant proteins and incorporated into the DNA vaccine did not possess the 22 N–terminal amino acids present in the proteins Smp–077310.2 and Smp–324770, nor the 4 N–terminal amino acids from the protein Smp–077310.1 (**Supplementary Figure 3B**). Additionally, our recombinant protein differs from the protein Smp–324770, where a substitution from a phenylalanine to a leucine in position 108 is observed (**Supplementary Figure 3B**).

A linear B cell epitope in the rSmtal–9 sequence was predicted by BepiPred (**Figure 3B**), but according to the predicted secondary structure of the protein obtained by computational modelling using Phyre2, most of the amino acids in this epitope (60–99) reside within a region that corresponds to an α–helix (**Figure 3C**), which is therefore likely to disrupt the linear B cell epitope. Additionally, NetMHCII predicted four H2–IAd–binding epitopes in rSmtal–9 and no H2–IAb–binding epitopes (**Figure 3B**). Thus, to perform the immunization protocols, C57BL–6 mice were used. The 3D structure of the recombinant protein (**Figure 3D**) demonstrates that the N–terminal part of the recombinant protein is rich in α–helices, while anti–parallel β–sheets can be observed in the C–terminal part of the protein (**Figure 3D**).

**Figure 4:**
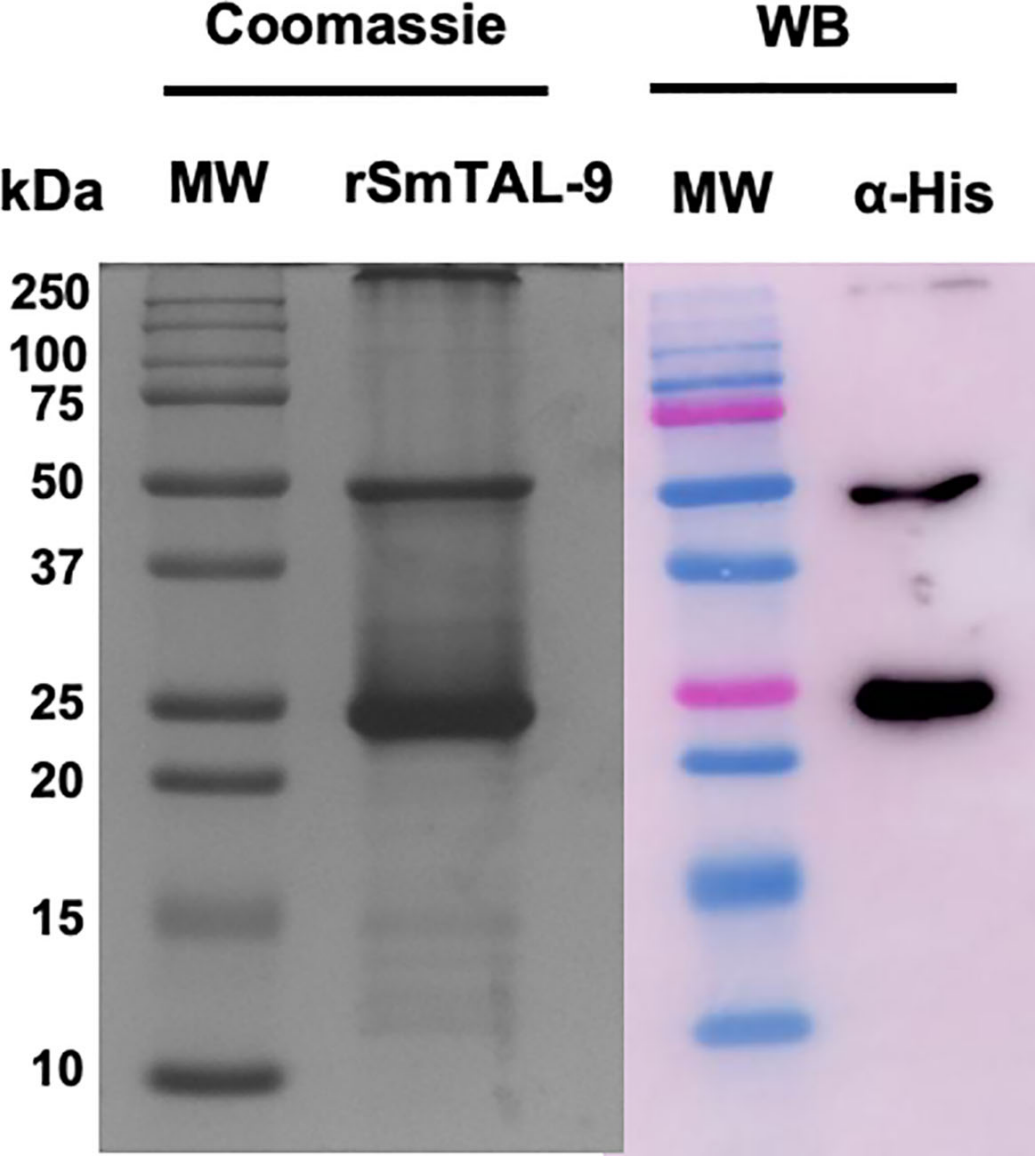
Electrophoretic and western blot analysis of the purified recombinant SmTAL–9. Purified rSmTAL–9 was detected using 15% SDS–PAGE stained with Comassie blue. Precision plus Protein™ Dual color standard from BioRad was used as the molecular weight standard (MW). Recognition of the 6XHis–tag from the recombinant protein was analyzed *via* western blotting using an anti–His (α–His) antibody.

The electrophoretic profile of the recombinant protein demonstrates a protein with approximately 25 kDa that can dimerize and migrate at 50 kDa in SDS–PAGE (**Figure 4**). Both the 25 kDa and 50 kDa bands are recognized by the anti–HIS antibody, thus indicating that it represents the recombinant protein that is expressed in fusion with 6X–His–tag (**Figure 4**).

Furthermore, our results using a prime–boost immunization protocol demonstrates that immunization of mice with Smtal–9 as antigen confers partial protection against infection with a significant reduction in parasite burden in mice (**Table 3**). The highest reduction in parasite burden (25.5% – 34.3%) was observed in mice that received a prime with the DNA vaccine containing the *Smtal–9 gene* and two boosts of a vaccine formulation with the recombinant protein. But even mice that received a prime with the control plasmid lacking the *Smtal–*9 sequence and two boosts with the recombinant protein showed a significant reduction in parasite burden (19.4% – 22.9%) (**Table 3**). Although immunization resulted in a significant decrease in worm burden, this reduction did not result in a significant reduction in the number of eggs trapped in the liver and intestine (**Table 3**).

**Table 3:** Protection induced by a prime (DNA vaccination) – boost (recombinant protein) regimen using SmTAL–9 as the antigen.

Immunization – Prime–Boost	Worm burden recovery^&^	Protectionlevel^a^	Egg/gram ofintestine^&^	Percentage reduction^b^	Egg/gram of liver^&^	Percentage reduction^b^
**Trial 1**						
pcDNA3.1V5/HIS + Saline/CFA/IFA	47 ± 10		8293 ± 3877		13678 ± 4941	
pcDNA3.1V5/HIS + rSmTAL–9/CFA/IFA	38 ± 7 (p = 0.044)*	19.4%*	7324 ± 2952 (p=0.8101)	11.7% ns	13060 ± 3511 (p > 0.999)	4.5% ns
pcDNA3.1V5/HIS/*SmTAL9 +* Saline/CFA/IFA	42 ± 9 (p = 0.665)	10.6% ns	8747 ± 4354 (p=0.8101)	0.0% ns	10720 ± 3940 (p = 0.2863)	21.62% ns
pcDNA3.1V5/HIS/*SmTAL9 +* rSmTAL–9/CFA/IFA	35 ± 7 (p = 0.0087)*	25.5%*	9611 ± 3636 (p=0.8101)	0.0% ns	13923 ± 3727 (p > 0.999)	0.0% ns
**Trial 2**						
pcDNA3.1V5/HIS + Saline/CFA/IFA	35 ± 6		9940 ± 3586		27777 ± 8902	
pcDNA3.1V5/HIS + rSmTAL–9/CFA/IFA	27 ± 11 (p =0.022)*	22.9%*	13810 ± 3752 (p=0.0195)*	0.0% ns	24208 ± 6800 (p > 0.999)	12.8% ns
pcDNA3.1V5/HIS/*SmTAL9 +* Saline/CFA/IFA	33 ± 6 (p = 0.502)	5.7% ns	11612 ± 4068 (p=0.3633)	0.0% ns	24720 ± 6470 (p > 0.999)	11.0% ns
pcDNA3.1V5/HIS/*SmTAL9 +* rSmTAL–9/CFA/IFA	25 ± 5 (p = 0.011)*	28.6%*	12815 ± 8324 (p=0.2998)	0.0% ns	31134 ± 9800 (p > 0.999)	0.0% ns

a Reduction in the total number of worms compared to pcDNA3.1V5/HIS + Saline/CFA/IFA control group.

b Reduction in the total number of eggs in tissue (liver and intestine) compared to pcDNA3.1V5/HIS + Saline/CFA/IFA control group.

^*^Statistically significant differences in comparison to pcDNA3.1V5/HIS + Saline/CFA/IFA control group.

^&^ Statistically significant differences were determined using Student’s t test or the Mann–Whitney U test.

## Discussion

A family of proteins that contains an N–terminal EF Hand motif and a C–terminal dynein light chain domain have been described in worm parasites was reviewed recently (21). In *S. mansoni*, this family of proteins have been denominated as *S. mansoni* tegument–allergen–like (Smtal) due to its location in the parasite tegument and its similarity with allergen (11, 12, 24, 25). The *S. mansoni* TAL family is composed of 13 members (10), and some have been associated with resistance to infection and reinfection (9, 14, 26).

Smtal–9 is a member of the TAL family which forms homodimers and can interact with Ca^2+^, thus suggesting its role is Ca^2+^–dependent signaling (15). Its expression is predicted to occur during all the parasite life cycle stages, but most abundantly in adult worms, eggs, and miracidia (10). Smtal–9 has been identified by our group during a proteomic study (unpublished data), in which this protein was recognized by sera from mice immunized with a preparation of the schistosomula tegument called Smteg (7). As immunization with Smteg induces a robust antibody response that transfers protection to a naïve recipient (16), we thus sought to evaluate the role of Smtal–9 in parasite development (*in vitro*) and survival (both *in vitro* and *in vivo*), as well as its ability to induce protection when used in a DNA prime–protein boost vaccination schedule.

Our RNAi assay demonstrated that the use of *Smtal–9*–dsRNA significantly reduced the levels of *Smtal–9* transcript in exposed schistosomula between days 4 to day 7 post–exposure reaching the striking level of reduction of 90%. The knockdown of *Smtal–9* expression resulted in a transient *in vitro* reduction in the schistosomula size in *Smtal–9*–dsRNA exposed parasites at day 4 post–exposure in comparison to both control groups (untreated parasites or *gfp*–dsRNA treated parasites) and at day 10 post–exposure in comparison to *gfp*–dsRNA–treated parasites. It is important to note that the Smtal family is composed of 13 members, and possible redundancy in their function might explain the transient modification observed in *Smtal–9*–dsRNA–treated parasites (27). For instance, besides Smtal–9, the expression of transcripts for *Smtal–1*, *Smtal–2*, *Smtal–8*, *Smtal–10*, *Smtal–12* and *Smtal–13* are all observed in 3 day and 6 day–schistosomula (10).

The exact role of proteins from the Smtal family is not known, but due to its dynein light chain domain, and the demonstration that Smtal–3, a member of the TAL family, can bind the *S. mansoni* dynein (SmDLC), it is speculated that these proteins may be involved in cytoskeletal process (12). Recently, it was demonstrated that Smtal–1 can bind the IQ motif of the voltage–gated calcium channel, called SmCa_v_1B, thus, suggesting a role for this protein in calcium homeostasis (28). In our *in vitro* assays, the knockdown of *Smtal–9* expression did not impact parasite survival, since no differences in parasite viability was observed between the different groups. In contrast, when confronted with the host immune system, there was a significant reduction in the number of parasites and eggs recovered from mice infected with *Smtal–9*–dsRNA–treated parasites. This outcome demonstrates that Smtal–9 is important for providing the parasite the means to deal with its host environment. To our knowledge, this is the first study to report the role of one member of the Smtal family in parasite survival. The discrepancy between our *in vitro* and *in vivo* assays could be explained by the lack of pressure in the *in vitro* system. This was also previously demonstrated for depleted parasites for other proteins described as essential for parasite development, such as some protein kinases (SmJNK, SmERK, Smp38, and SmFES). For instance, these parasites presented relevant phenotypic alterations after confrontation with the host immune system, ranging from lack of worm maturation, underdeveloped internal structures, and reduced numbers of parasites, among other changes (28–30).

Thus, the impact of the *Smtal–9* expression knockdown on parasite survival *in vivo*, in addition to the fact that the Smtal–9 sequence possesses predicted B and T cell epitopes, suggests this antigen is a potential vaccine candidate. Indeed, the use of this antigen in our prime–boost vaccine schedule, induced a significant reduction in parasite burden in Smtal–9 immunized mice. But this formulation did not impact egg load in the liver or in the intestine. Although our results validate Smtal–9 as a vaccine candidate (proof of concept), better vaccine formulation still need to be developed to improve the protective immunity induced by this antigen. Then, by improving the effect of the vaccine on worm burden is expected to also observe a significant reduction in egg load. In this regard, the use of other adjuvants, optimization of the antigen obtention process and the use of better delivery systems are alternatives to improve vaccine efficacy. Also, a better understanding of the immune mechanisms involved in parasite elimination upon vaccination with Smtal–9 is important to drive the design of a better vaccine.

How the immune response triggered by vaccination acts in parasite elimination is puzzling. Some evidence suggests that antibodies would play an important role in the parasite elimination upon vaccination with Smtal–9: (i) in an immune proteomic assay, Smtal–9 is recognized by sera from mice immunized with a preparation of the schistosomula tegument (Smteg) (data not shown), a vaccine formulation that induces a protective immune response in mice (5), (ii) sera from Smteg–immunized mice can transfer protection to a naïve recipient (16). However, our *in silico* analysis of the protein sequence predicts it has a cytoplasmic location inaccessible to antibodies.

Therefore, what would be the importance of antibodies in the protective immune response since it would seem unlikely to be able to bind its target in live parasites? Data from a recently published proteomic study helps to clarify this question, as Smtal–9 was identified as one of the proteins associated with the *S. mansoni* tegument membrane of the schistosomula (31). This observation suggests that this antigen is exposed to host immune response, and, thus, could be recognized by antibodies produced upon immunization.

The same study also demonstrates that Smtal–9 expression increases during larval maturation, with higher expression levels being observed in 5–day–old parasites (31). Previous observations have demonstrated that schistosomes become refractory to antibody–dependent cellular and complement–mediated damage as they migrate from skin to the lungs (31–33). Thus, we hypothesize that if antibodies play a role in the immune response induced by vaccination with Smtal–9, parasite–killing mechanisms may involve antigen functional blockage rather than an Fc–effector–mediated–killing of the parasite. But this hypothesis, as well as the role of antibody in Smtal–9–induced protective immunity need to be proven by further studies.

Regardless of the mechanism involved in parasite elimination upon vaccination with Smtal–9 antigen, our data demonstrate that Smtal–9 is a promising antigen to be used in a vaccine formulation against schistosomiasis. We also demonstrated, for the first time, that one of the TAL family members, Smtal–9, plays an important role in parasite survival in its definitive host, although its function during parasite development and survival still needs to be determined.

## Data Availability Statement

The original contributions presented in the study are included in the article/**Supplementary Material**. Further inquiries can be directed to the corresponding author.

## Ethics Statement

The animal study was reviewed and approved by Ethics Committee of Animal Use of the Fundação Oswaldo Cruz (FIOCRUZ) under licenses LW26/12 and LW22/16.

## Author Contributions

WB performed the experiments and the data analysis, discussed the data and wrote the manuscript, ID performed the experiments and the data analysis, discussed the data and revised the manuscript, RS–P contributed to the design of the research, performed the experiments, discussed the data and revised the manuscript, MM contributed to the design of the research, provided some reagents, discussed the data and revised the manuscript and CF designed the research, obtained funding, performed the experiments and the data analysis, discussed the data and wrote the manuscript. All authors approved the submitted version.

## Funding

This study was financed in part by the Coordination for the Improvement of Higher Education Personnel (Coordenação de Aperfeiçoamento de Pessoal de Nível Superior–CAPES) – Finance Code 001, Conselho Nacional de Desenvolvimento Científico e Tecnológico–Brasil (Grant Nos. 407702/2012–1, 303131/2018–7 and 317389/2021–1), Instituto René Rachou – FIOCRUZ–MG, Programa CAPES/Print/FIOCRUZ and Rede De Pesquisa em Imunobiológicos do Estado de Minas Gerais – FAPEMIG (RED00140–16). Fellowships: Pq–CNPq (CF and MM).

## Conflict of Interest

The authors declare that the research was conducted in the absence of any commercial or financial relationships that could be construed as a potential conflict of interest.

## Publisher’s Note

All claims expressed in this article are solely those of the authors and do not necessarily represent those of their affiliated organizations, or those of the publisher, the editors and the reviewers. Any product that may be evaluated in this article, or claim that may be made by its manufacturer, is not guaranteed or endorsed by the publisher.
