# Supplementary figures and images for: SmTAL-9, a Member of the Schistosoma mansoni Tegument Allergen-Like Family, Is Important for Parasite Survival and a Putative Target for Drug/Vaccine Development
Source: Front Immunol. 2022 Jul 12;13:889645. doi: 10.3389/fimmu.2022.889645 (PMC9336510; doi:10.3389/fimmu.2022.889645)

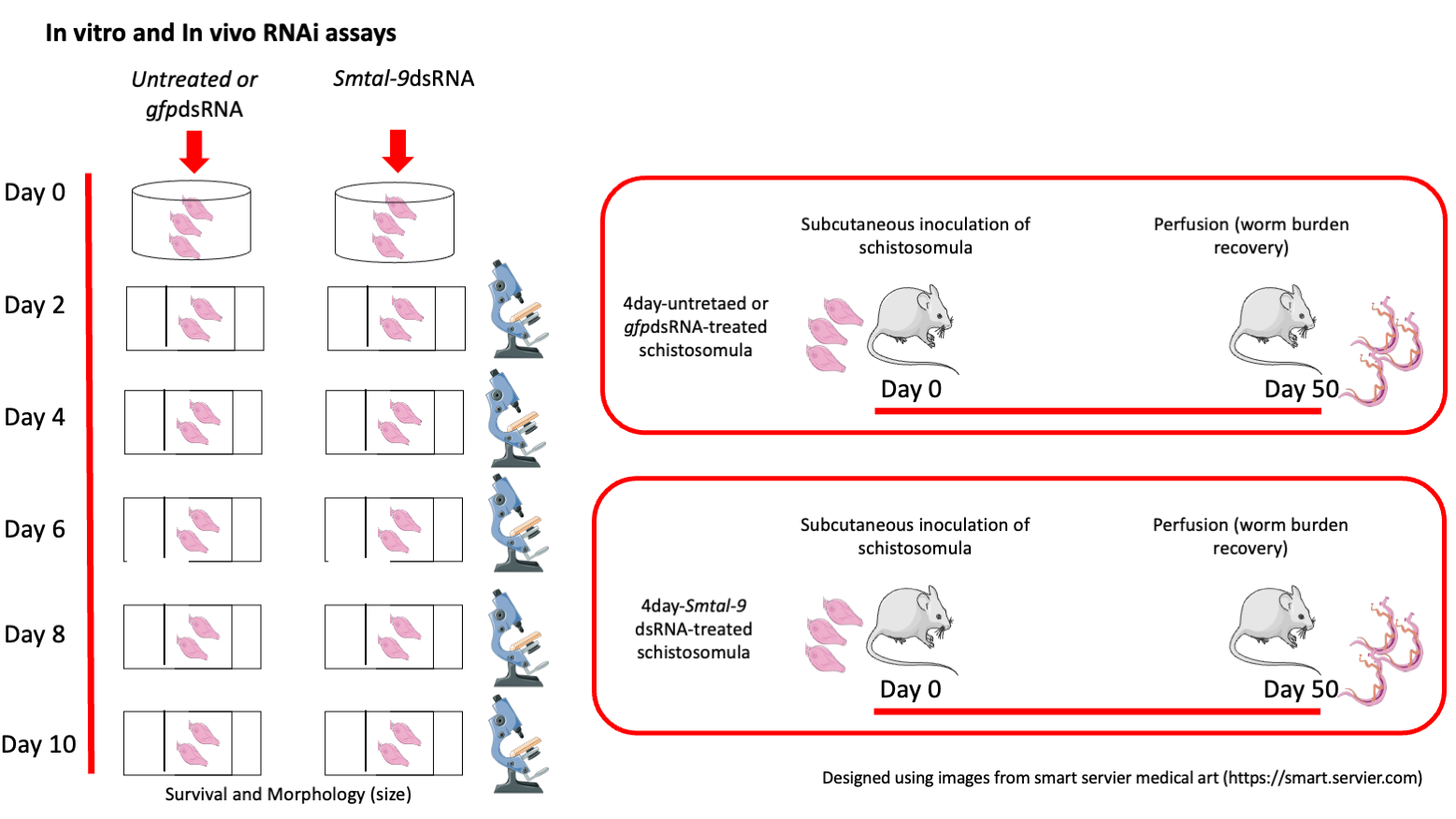

Supplement: Supplementary file 1 [file Image_1.tiff]

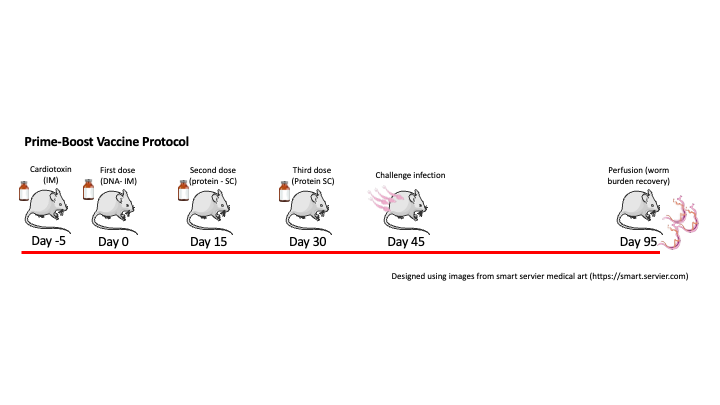

Supplement: Supplementary file 2 [file Image_2.tiff]

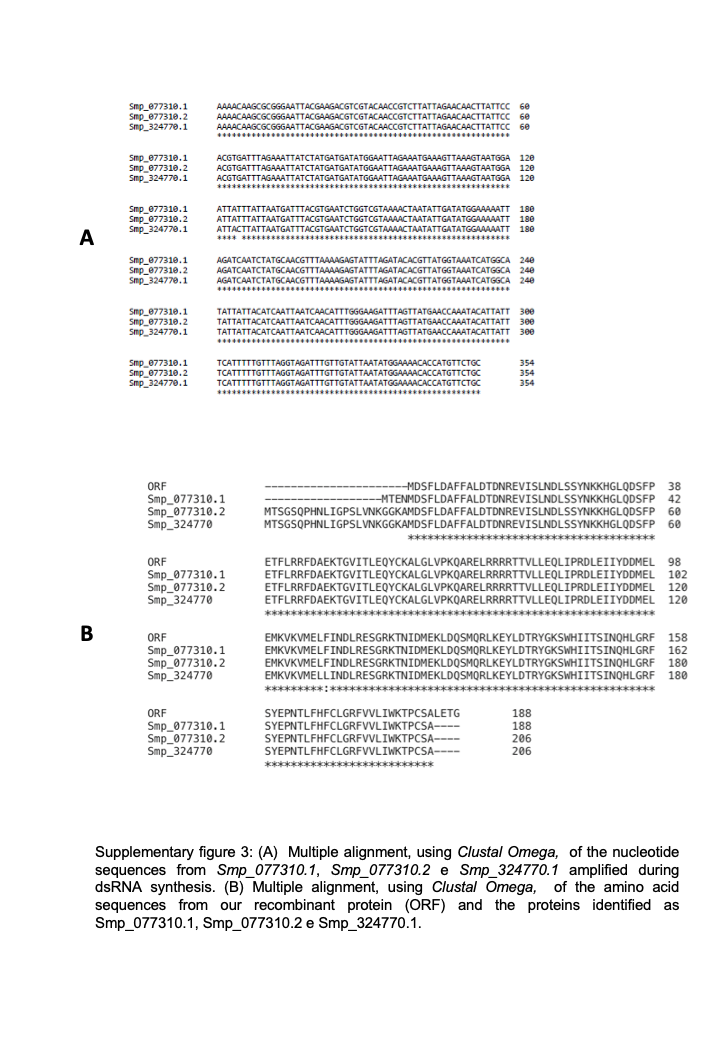

Supplement: Supplementary file 3 [file Image_3.tiff]
